# Comparison of Esterman disability scores obtained using Goldmann perimetry and the Humphrey field analyzer in Japanese low-vision patients

**DOI:** 10.1371/journal.pone.0203258

**Published:** 2018-09-13

**Authors:** Mieko Yanagisawa, Satoshi Kato, Makiko Ochiai

**Affiliations:** Department of Ophthalmology, University of Tokyo Graduate School of Medicine, Tokyo, Japan; University of California San Diego, UNITED STATES

## Abstract

**Purpose:**

To compare the Esterman Disability Score (EDS) obtained with Goldmann perimetry (GP) testing and the Humphrey field analyzer (HFA) in low vision Japanese subjects. Subjects were also divided into groups by diagnosis to examine how disease influences EDS measurements.

**Methods:**

The EDS was obtained using GP (GP-EDS) and the built-in testing program of the HFA (HFA-EDS). Tests were performed within 3 months of each other. Regression analyses were used to examine the relationship between GP-EDS and HFA-EDS.

**Results:**

A total of 128 visually impaired subjects were included in this study. Subjects had low vision because of glaucoma (57 subjects), age-related macular degeneration (AMD, 17 subjects), retinitis pigmentosa (RP, 17 subjects), and other causes (37 subjects). The GP-EDS obtained was well-correlated with HFA-EDS (r = 0.87, P < 0.001) and it was possible to estimate HFA-EDS from GP-EDS. The GP-EDS was significantly lower than the HFA-EDS in eyes with glaucoma and RP. There was no significant difference between EDS values in eyes with AMD or other disease.

**Conclusion:**

The GP-EDS correlated well with the HFA-EDS. However, the relationship between the EDS measured with the two different testing modalities varies by disease.

## Introduction

The extent of visual field (VF) impairment widely varies and is dependent upon which region is affected and the severity of the VF impairment that has occurred. In some cases, VF impairment considerably limits movement and the ability to walk, inevitably leading to a deterioration in vision-related quality of life (VRQOL).

Previous studies have reported that both VRQOL and health-related QOL decreases with increasing VF impairment severity[1,2]. Difficulties performing everyday activities vary and are heavily dependent upon the functioning portions of the VF[3,4]. Therefore, VF testing is important for understanding the degree of VF impairment. The VF is usually tested with static or dynamic perimetry. In static perimetry, results are directly fed from an automated perimeter into a computer, enabling objective examination and analyses. Dynamic perimetry is performed using a Goldmann perimeter (GP), which provides results that are drawn on an examination sheet. These are then subjectively evaluated by a perimetry specialist. Dynamic perimetry has the limitations of requiring a specialist to interpret test results and it only provides qualitative VF information. Therefore, it is difficult to use dynamic perimetry to determine how much and how fast a VF defect is progressing. Static perimetry is able to overcome these limitations of dynamic perimetry[5,6].

Goldmann perimetry VF results are often used in Japan to qualify patients for a formal visually impaired certification. As previously proposed by the American Medical Association (AMA), the sum of angles of an existing VF on 8 meridians in the GP map is used to evaluate VF impairment (AMA scale)[7]. However, this method has several problems, including what is considered a “normal VF area.” The normal area was originally established as a wide range, and “the ratio of VF impairment,” calculated using the I-2e isopter, is generally reduced in both visually impaired and non-impaired individuals. Furthermore, position differences of VF defects are not considered important and, with this evaluation method, upper hemianopia and lower hemianopia are noted in the same manner.

Esterman noticed another limitation of the AMA scale. Mainly, central vision and lower hemianopia were not weighted properly in the overall score. Therefore, Esterman developed his own scoring system, which is now known as the Esterman disability score (EDS)[8–11]. Esterman validated his scoring method in a study that included 2000 glaucoma patients and, in 1984, the AMA adopted the EDS as the standard for evaluating visual impairment[12]. The AMA now also uses the Functional Vision Score (FVS)[13], which differs from the EDS in the arrangement of the evaluation grid used to calculate the final score. The EDS grid does not include the central 3 degrees of vision because the central VF is calculated from visual acuity measurements. However, the FVS grid places half of all regions within the central 10 degrees of vision. Where the EDS mainly evaluates peripheral VF, the FVS also includes the central VF.

The official visual impairment grade is determined in Japan using visual acuity measurements and VF impairment severity. Therefore, it is important for Japanese low vision individuals to include peripheral VF examinations when evaluating VF impairment. We consider the EDS to be more suitable for evaluating VF impairment than either the AMA or FVS scales. Furthermore, the EDS was recently made the official method for evaluating VF impairment in Japan. The current study compares the EDS scores obtained with GP and HFA in Japanese patients. Study subjects were also subdivided by disease type to better understand how disease influences the correlation between GP and HFA EDS measurements.

## Methods

This study was conducted at the Department of Ophthalmology at the University of Tokyo Graduate School of Medicine. The study protocol was reviewed and approved by the University of Tokyo Hospital Institutional Review Board and all study conduct adhered to the tenets of the Declaration of Helsinki. Informed consent was obtained from all patients after the study purpose, experimental procedures, and risks/benefits of study participation were carefully and thoroughly explained.

All subjects underwent GP at the University of Tokyo Hospital ophthalmology clinic between June 2015 and February 2016. Subjects were considered for inclusion in this study if all of the following were true: (1) measurements with the Goldmann III-4e isopter could be obtained, (2) they had undergone EDS testing with the HFA within 3 months of enrollment, and (3) they met the VF impairment criteria using the official Japanese visual impairment grading system.

**Fig 1:**
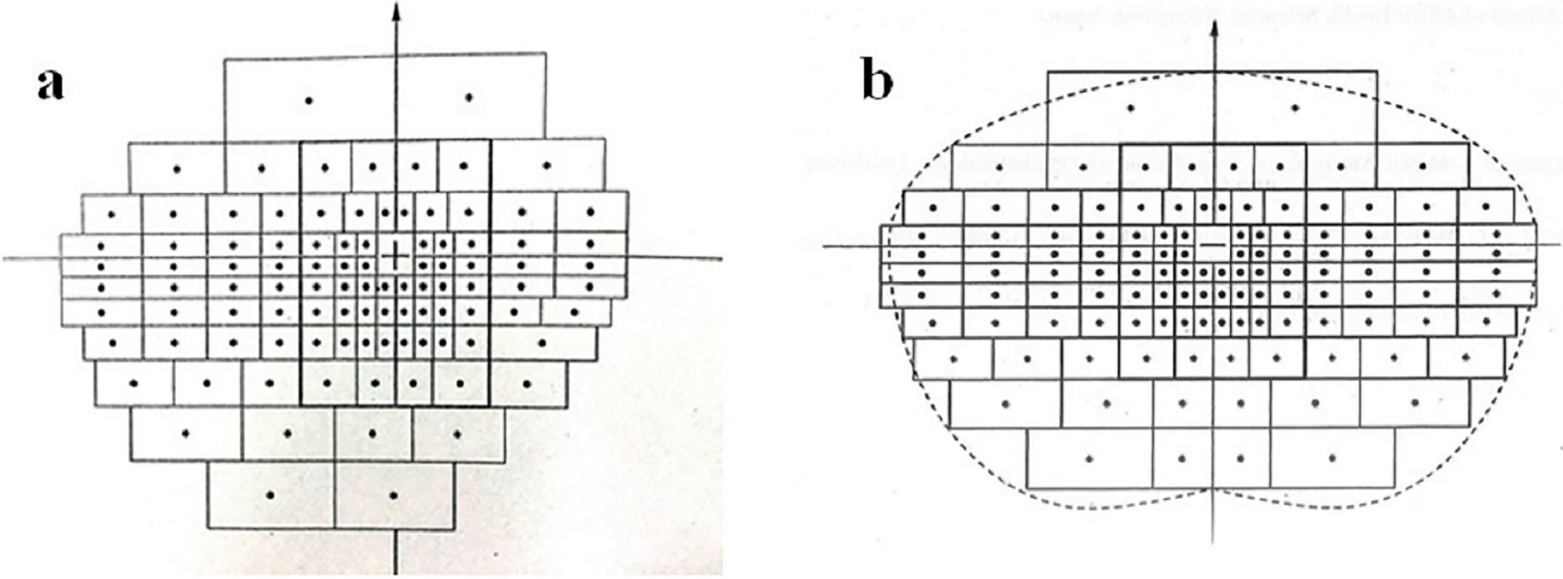
Esterman grids for Goldmann perimetry. a: Unilateral Esterman grid. b: Unilateral or bilateral Esterman grid.

**Fig 2:**
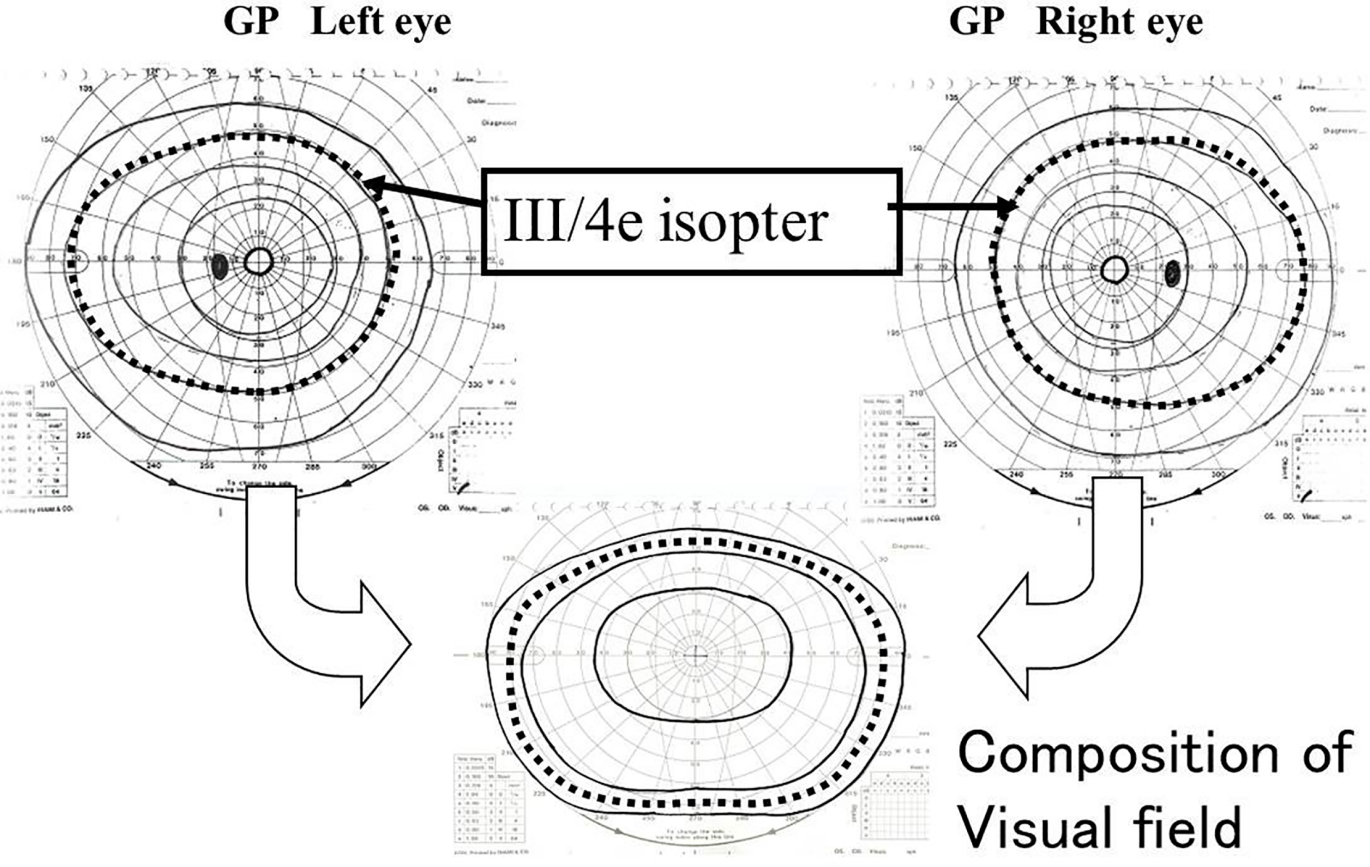
Binocular visual fields were measured on a composite of visual fields obtained from both eyes with Goldmann III-4e isopter perimetry. The absolute blind spot was evaluated as visual field loss in both eyes.

**Fig 3:**
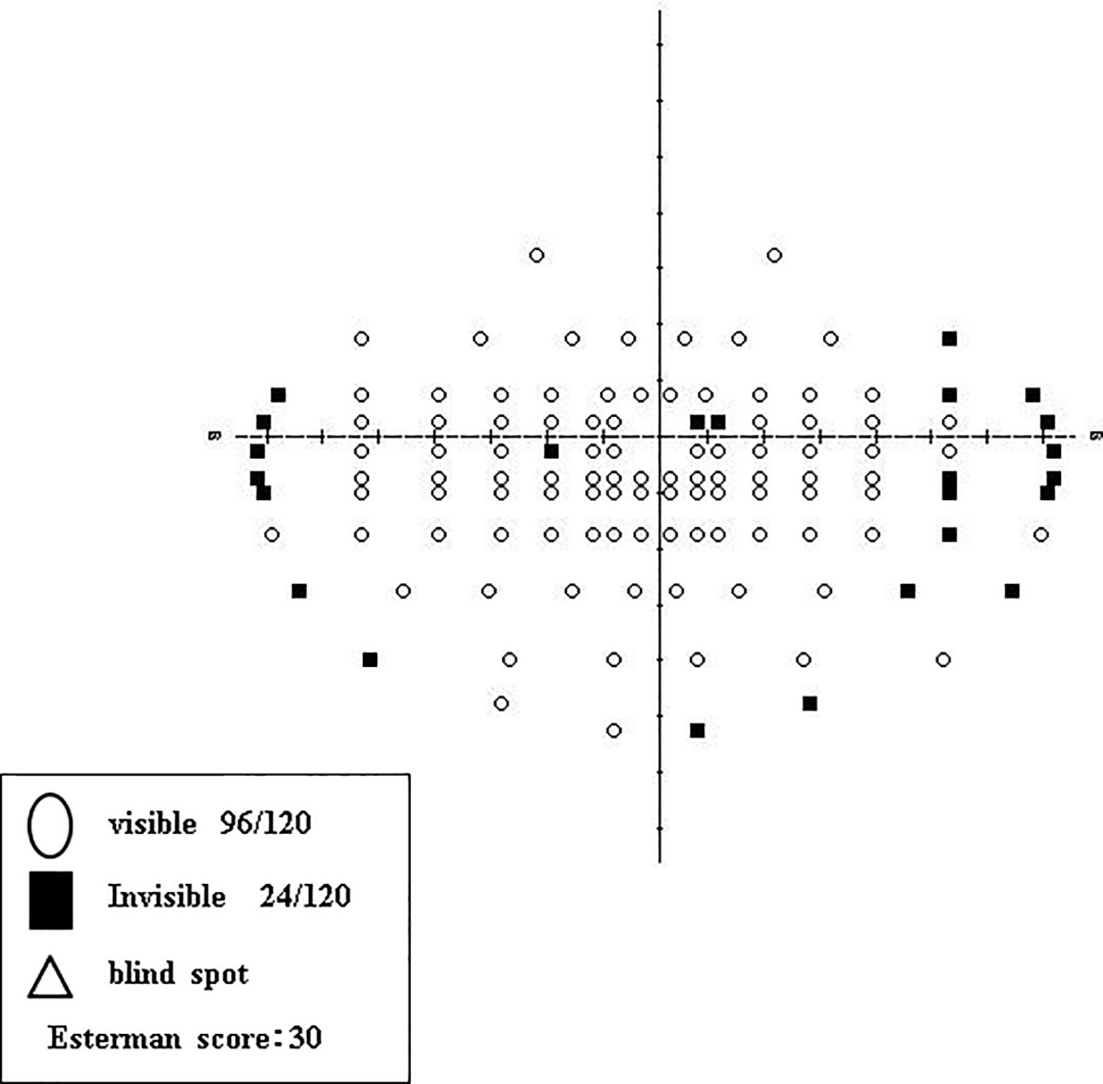
Results of the Humphrey field analyzer built-in “Esterman” program.

The EDS was calculated from GP measurements using the Esterman grid for dynamic perimetry (Fig 1). If a patient was blind in one eye, the dynamic perimetry EDS score was calculated by counting dots included in the III-4e isopter using tracing paper printed with an Esterman grid (Fig 1A). However, we did not count dots on isopter lines, within a scotoma, or on a scotoma boundary line. The total number of dots on the grid was 100 and the counted dots were taken as the EDS (%). If the VF in both eyes remained intact, the VF was estimated using a composite measurement of the VF in both eyes for the GP III-4e isopter test. The composite VF was obtained by overlaying the result sheet from both eyes and creating a VF range (Fig 2). We then counted the dots included in the III-4e isopter dynamic perimetry test and used an Esterman grid on tracing paper to draw on dots for both eyes (Fig 1B). Because the maximum number of dots for this grid was 120, the EDS was calculated by dividing the number of counted dots by 120 and multiplying by 100 to obtain a percentage. The Humphrey Field Analyzer (HFA) EDS was evaluated using the “Esterman” program that is built-in to the HFA system (Carl Zeiss Dublin, CA, USA). Testing results were printed out for further analysis (Fig 3).

### Data analyses

Data are presented as mean ± standard deviation where applicable. A comparison between EDS values obtained with GP (GP-EDS) and HFA (HFA-EDS) was made in Japanese subjects with visual impairments. Subjects were striated by disease and the correlation between GP-EDS and HFA-EDS values was re-examined using Spearman correlation coefficients. Regression and residual analyses of GP-EDS and HFA-EDS were also performed. All statistical analyses were performed using PASW Statistics (version 17.0, SPSS, Inc., Chicago, IL, USA) and statistical significance was defined as P < 0.05.

## Results

A total of 128 consecutive Japanese subjects (76 [59.0%] men, 52 [41.0%] women) with a mean subject age of 63.4 ± 15.1 years (range, 24–91 years) were included in this study. Nearly all patients had a stable VF and had been certified as visually impaired individuals in Japan. The causes of vision loss were glaucoma (n = 57 [44.5%] subjects, age-related macular degeneration (AMD, n = 17 [13.3%] subjects), retinitis pigmentosa (RP, n = 17 [13.3%] subjects), corneal disease (n = 10 [7.8%] subjects), diabetic retinopathy (DR, n = 9 [7.0%] subjects), or other causes (n = 18 (14.1%) subjects).

**Fig 4:**
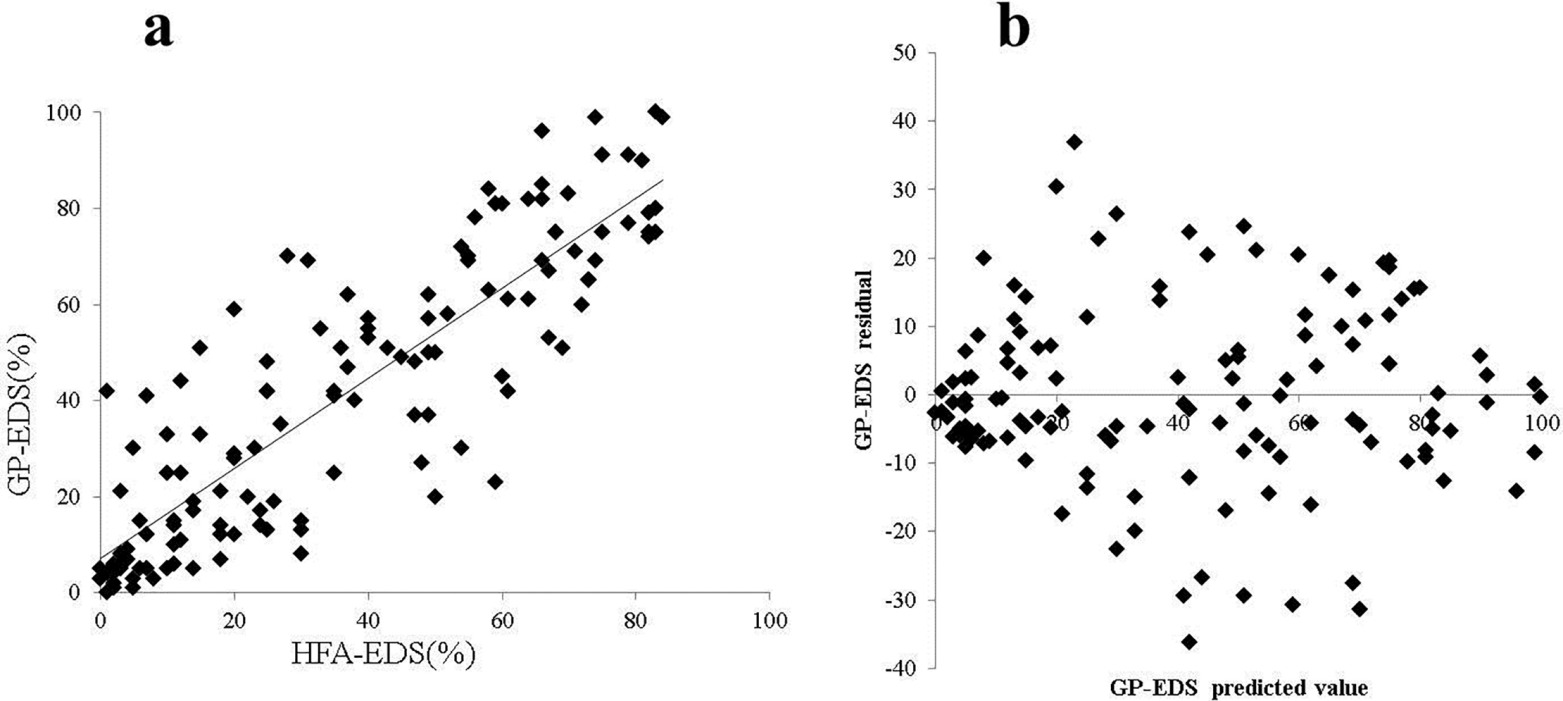
Regression plot of GP-EDS and HFA-EDS (a) and the residual distribution (b).

There was a significant correlation between GP-EDS and HFA-EDS when all subjects were examined (r = 0.87, P < 0.001; Fig 4A). The regression equation for this relationship (R^2^ = 0.75) was:

GP−EDS=0.8075×HFA−EDS+4.0

We also performed a residual analysis to investigate the validity of the regression equation. The residual distribution for this correlation was random (Fig 4B).

**Fig 5:**
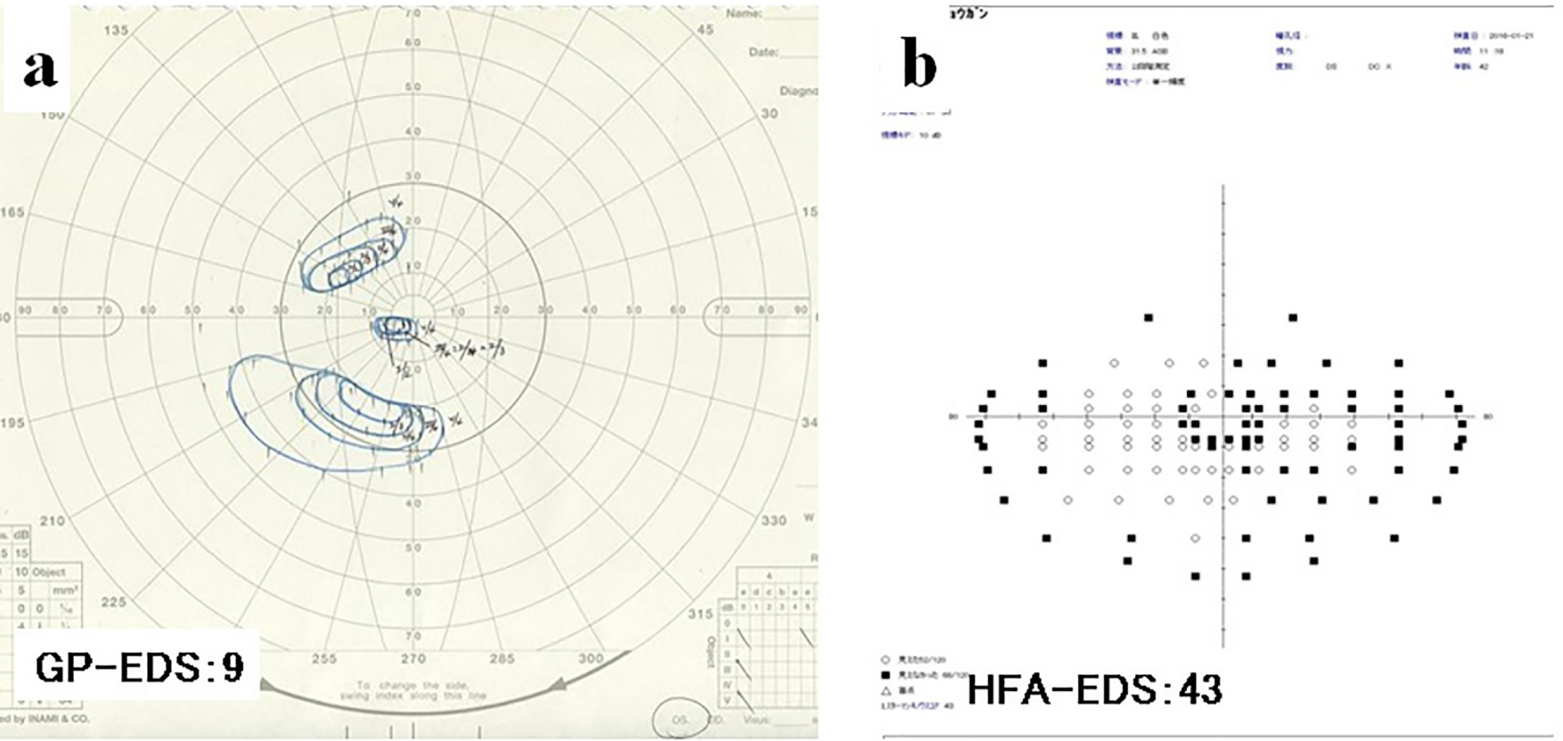
A representative case of a subject with glaucoma.

Subjects with glaucoma had a GP-EDS and HFA-EDS of 35.7 ± 26.0 and 44.0 ± 27.6, respectively, a difference that was statistically significant (P = 0.010, Fig 5). The GP-EDS (14.2 ± 21.4) and HFA-EDS (23.6 ± 29.3) were also significantly different in subjects with RP (P = 0.004). The GP-EDS and HFA-EDS were not significantly different from each other in subjects with AMD (GP-EDS: 67.5 ± 12.1, HFA-EDS: 65.0 ± 21.4; P = 0.38), corneal disease (GP-EDS: 26.7 ± 10.7, HFA-EDS: 21.5 ± 15.7; P = 0.15), DR (GP-EDS: 41.6 ± 25.5, HFA-EDS: 39.6 ± 29.7; P = 0.69, or other causes of visual impairment (GP-EDS: 44.4 ± 23.9, HFA-EDS: 42.8 ± 26.6; P = 0.67).

## Discussion

Here, we evaluated VF impairments of low vision, Japanese subjects using the EDS measured with GP and HFA. Patients were also examined by disease. In all cases, GP-EDS and HFA-EDS were significantly correlated with each other and GP-EDS can be sufficiently calculated from the HFA-EDS value. Interestingly, GP-EDS was significantly lower than HFA-EDS in glaucoma and RP subjects. There was no significant difference between EDS measurements any other disease group (cornea disease, AMD, DR, and other disease). However, in subjects with concentric contraction of the VF, GP-EDS was significantly smaller than HFA-EDS. Glaucoma patients also had a significantly smaller GP-EDS than HFA-EDS. Unfortunately, the HFA does not evaluate fixation and when fixation is poor, EDS will be high.

The EDS has been previously shown to be correlated with a variety of disabilities due to visual impairment in glaucoma patients. All studies[14,15], investigated the correlation between everyday living capabilities and EDS using a questionnaire focused on daily activities. Significant correlations were identified for reading, of character, walking collisions, stumbling, using stairs, and finding fallen objects. Furthermore, Gezer et al[16] examined HFA-EDS in 34 functional amblyopic patients and 10 normal subjects and found a significant difference in their scores.

There are 2 ways to obtain the EDS. One method uses dynamic perimetry and manual calculations, while the other method uses static perimetry and automated software calculations. Both methods have advantages and disadvantages. In dynamic perimetry, EDS is calculated from a binocular VF that is created using a composite of VF measurements from both eyes. However, static perimetry stimuli better mimic everyday life visual stimuli. The time it takes to calculate EDS is shorter for static perimetry than for dynamic perimetry. Lastly, when VF impairments are advanced, especially when central vision is poor, fixation losses can influence static perimetry results. In these cases, examination with dynamic perimetry is more suitable than examination with static perimeter. Patients are required to place their chin in the center of the chin support during HFA measurements. Therefore, patient fixation cannot be checked in the monitor. In summary, poor central visual acuity, concentration losses, and comprehension problems all make dynamic perimetry a better choice than static perimetry.

The correlation between GP-EDS and HFA-EDS is somewhat controversial. Yamagata et al[17] reported that the relationship between them can be described by the following regression equation (R^2^ = 0.72):

GP−EDS=0.89×HFA−EDS+8.08

However, Colenbrander et al[18] reported only a poor correlation between GP-EDS and HFA-EDS. They actually recommended that the functional field score (FFS) be used to evaluate VF impairment and not EDS because FFS more heavily weights the central visual field. The EDS calculation grid does not have a specific central visual field region and it does not include isopter lines. As a result, we found that when the remaining functional VF was small, EDS was low. Thus, FFS may be more suitable than EDS for evaluating patients with concentric contraction of the VF (e.g., glaucoma and RP patients).

Langelaan et al[19] investigated field loss underestimation with FFS in visually impaired patients with Goldmann isopters III-4e and V-4e. This was done to develop a predictive model for FFS(III-4e) using FFS(v-4e) values. The model was adjusted for possible confounders. Results showed that FFS(V-4e) was consistently higher than FFS(III-4e), with a mean difference of 14.56 points (95% CI: 12.48–16.64). Multiple linear regression analysis showed that age, functional acuity score, primary eye disease, and central-peripheral loss did not confound predictions of FFS(III-4e) and that FFS(III-4e) could be estimated with the following equation:

FFS(III−4e)=1.063×FFS(V−4e)−19.25

Therefore, simply subtracting 19.25 points from the FFS(V-4e) value was sufficient for estimating FFS(III-4e). Based on our regression analysis, GP-EDS can be estimated by adding 4.0 points to the HFA-EDS value.

In conclusion, the relationship between GP-EDS and HFA-EDS is linear and HFA-EDS can be used to estimate GP-EDS. In practice, simply adding 4.0 points to the obtained HFA-EDS value will be sufficient for estimating GP-EDS. This result increases our confidence in using the HFA to quantify VF impairment. However, the influence of disease-related VF contraction, as in glaucoma and RP, should not be ignored.

## Supporting information

S1 Data (XLS)
